# Pulmonary veno-occlusive disease after immune checkpoint inhibitor therapy: an autopsy case report

**DOI:** 10.1093/ehjcr/ytag459

**Published:** 2026-06-16

**Authors:** Saki Hasegawa-Tamba, Tsugumi Sato, Takahide Arai, Shintaro Nakano

**Affiliations:** Department of Cardiology, International Medical Center, Saitama Medical University, 1397-1 Yamane, Hidaka, Saitama 350-1298, Japan; Division of Pathology, International Medical Center, Saitama Medical University, 1397-1 Yamane, Hidaka, Saitama 350-1298, Japan; Department of Cardiology, International Medical Center, Saitama Medical University, 1397-1 Yamane, Hidaka, Saitama 350-1298, Japan; Department of Cardiology, International Medical Center, Saitama Medical University, 1397-1 Yamane, Hidaka, Saitama 350-1298, Japan

**Keywords:** Immune checkpoint inhibitors, Pulmonary veno-occlusive disease, Immune-related adverse events, Case report

## Abstract

**Background:**

Immune checkpoint inhibitors, including nivolumab and ipilimumab, have revolutionized cancer therapy by enhancing immune-mediated antitumour activity. While some cardiovascular immune-related adverse events manifest as various phenotypes, such as myocarditis and pericarditis, pulmonary veno-occlusive disease is a particularly rare complication.

**Case summary:**

An 80-year-old man with chronic obstructive pulmonary disease, hypertension, and a history of heavy smoking underwent lobectomy for lung cancer. Histopathological examination of pulmonary tissue, including the vasculature, showed no evidence of pulmonary veno-occlusive disease at the time of surgery. Following disease recurrence, he received combination therapy with nivolumab and ipilimumab. Shortly after treatment initiation, he developed respiratory failure and a renal immune-related adverse event, which improved with oral corticosteroid therapy. Ipilimumab was discontinued due to its higher risk of severe immune-related adverse events, and nivolumab monotherapy was continued for 22 months until the 14th cycle, when the patient presented with progressive dyspnoea and severe hypoxaemia. Echocardiography and right heart catheterization confirmed pulmonary hypertension. Pulmonary vasodilator therapy was initiated, but the respiratory failure rapidly progressed, and the patient died on hospital day 53. Autopsy revealed intimal fibrous thickening and smooth muscle proliferation of interlobular pulmonary veins, consistent with pulmonary veno-occlusive disease.

**Discussion:**

This case highlights pulmonary veno-occlusive disease as a rare but potentially fatal pulmonary vascular complication associated with immune checkpoint inhibitor therapy. In patients receiving immune checkpoint inhibitors who develop unexplained pulmonary hypertension and severe hypoxaemia, pulmonary veno-occlusive disease should be considered as a differential diagnosis.

Learning points

- Immune checkpoint inhibitors (ICIs) have revolutionized cancer treatment but may contribute to pulmonary vascular complications in rare cases.
- Close monitoring is essential for patients receiving ICI therapy.
- In patients on ICI therapy who develop unexplained pulmonary hypertension and hypoxaemia, pulmonary veno-occlusive disease should be considered as a differential diagnosis.

## Introduction

Immune checkpoint inhibitors (ICIs), including nivolumab and ipilimumab, have revolutionized cancer therapy by enhancing immune activity through the blockade of T-cell inhibitory receptors. While these agents provide substantial antitumour efficacy, they can also induce immune-related adverse events (irAEs) affecting multiple organ systems.^1^

While some cardiovascular irAEs manifest as a wide range of phenotypes, pulmonary veno-occlusive disease (PVOD) is a particularly rare complication.^2–4^ Most reported cases of pulmonary vascular involvement have described pre-capillary pulmonary arterial hypertension (PAH).^5,6^ In contrast, only one published report has documented the occurrence of PVOD in one of 42 patients with ICI-associated pulmonary hypertension (PH); however, the clinical course was not described.^4^

We report a case of PVOD that became clinically apparent after approximately 2 years of ICI therapy for lung cancer. Differentiation from pre-capillary PAH is important because PVOD requires cautious management with pulmonary vasodilators. This report underscores the need for clinicians to consider PVOD as a potential, although rare, complication of ICI therapy.

## Summary figure

**Figure:**
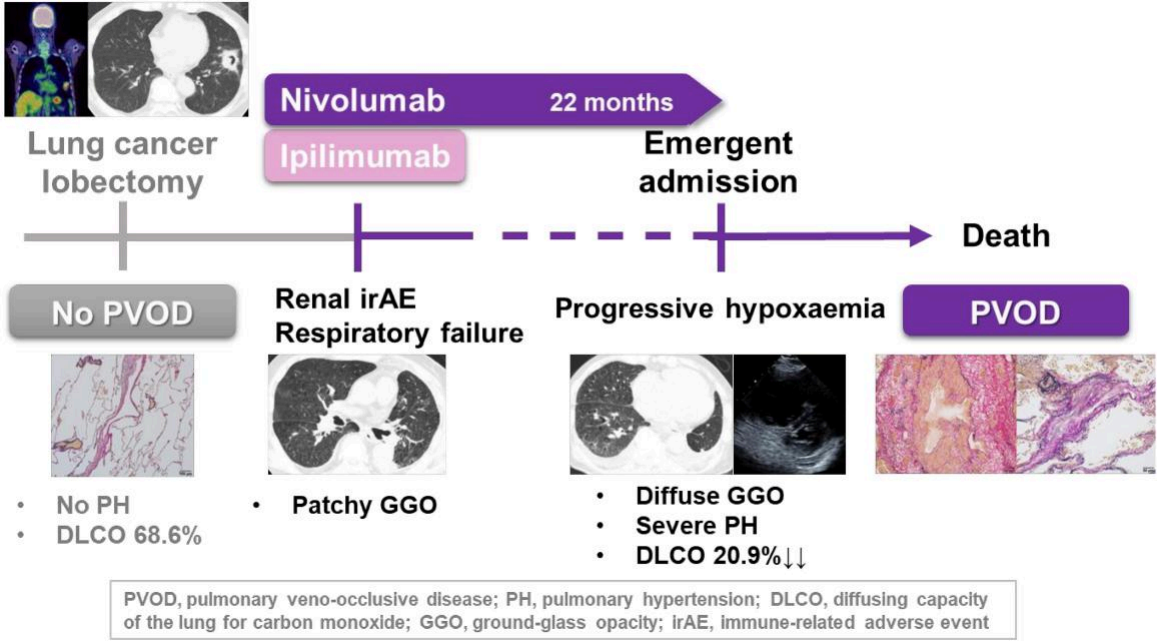


## Case presentation

**Figure 1:**
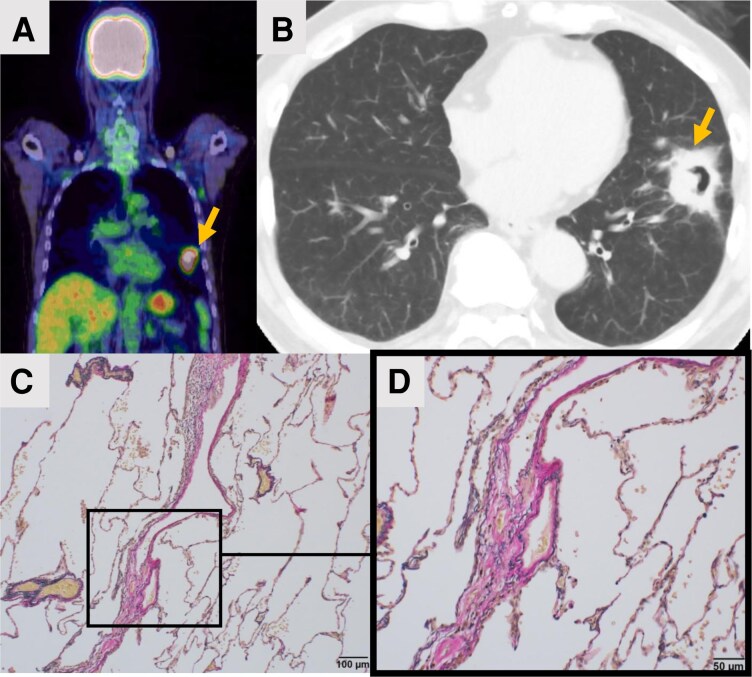
Findings at the time of lobectomy. (*A*) Fluorodeoxyglucose positron emission tomography and (*B*) computed tomography show a lung cancer in the left lower lobe (arrow). (*C*, *D*) Histological examination of the resected lung tissues shows no abnormalities in the pulmonary veins. Sections stained with Elastica van Gieson.

**Figure 2:**
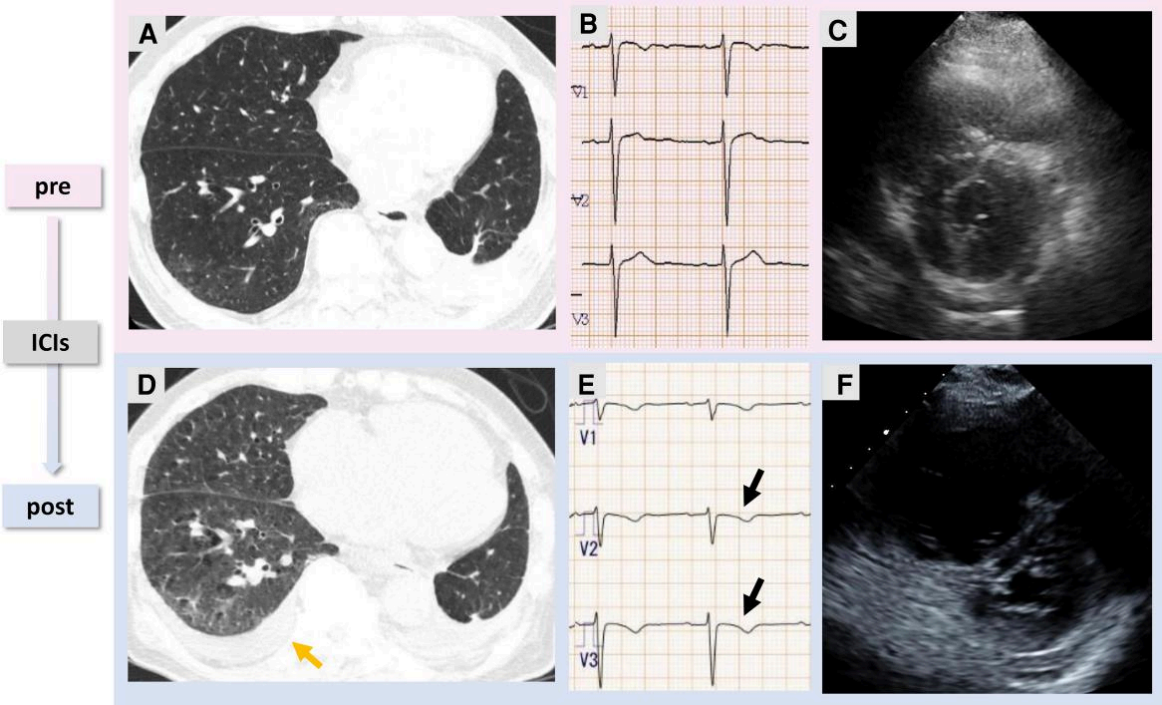
Imaging findings before and after initiation of immune checkpoint inhibitors (ICIs) therapy. (*A*) Chest computed tomography (CT), (*B*) electrocardiography, and (*C*) transthoracic echocardiography prior to ICIs therapy are unremarkable. (*D*) Chest CT images obtained at the time of emergency admission following the 14th cycle of nivolumab monotherapy show bilateral diffuse ground-glass opacities and pleural effusions (yellow arrow), along with right-sided cardiac chamber enlargement. (*E*) Electrocardiography reveals T-wave inversions in leads V1 to V3 (black arrows), and (*F*) echocardiography shows a D-shaped left ventricle, indicating right ventricular pressure overload.

An 80-year-old man with chronic obstructive pulmonary disease, hypertension, and a history of heavy smoking was emergently admitted with worsening dyspnoea. Three years earlier, he had been diagnosed with stage II left lower lobe lung cancer and had undergone lobectomy (*Figure 1A, 1B*). Histological examination of the resected lung confirmed squamous cell carcinoma and emphysematous changes, without abnormalities in the peripheral pulmonary arteries or veins (*Figure 1C, 1D*). Postoperative chest computed tomography (CT) revealed no additional abnormalities apart from the emphysematous changes (*Figure 2A*). Electrocardiography was unremarkable, and transthoracic echocardiography showed no findings suggestive of PH, including no right ventricular dilatation or interventricular septal flattening (*Figure 2B, 2C*).

During postoperative follow-up, recurrence involving the mediastinal lymph nodes and pleura was suspected, leading to the initiation of combination therapy with nivolumab (360 mg, fixed dose) and ipilimumab (74.7 mg, 1 mg/kg). One week after the first cycle, the patient developed fever, acute kidney injury, and respiratory failure. He was diagnosed with grade 1 acute interstitial nephritis, which improved with oral corticosteroid therapy (prednisolone 20 mg daily). Chest CT revealed bilateral, patchy ground-glass opacities. Respiratory failure improved with diuretics, but home oxygen therapy was required due to exertional hypoxaemia. Ipilimumab was discontinued due to its higher risk of severe irAEs, and nivolumab monotherapy was continued and achieved regression of the lung cancer. No other chemotherapeutic agents were administered.

One week after the 14th cycle of nivolumab monotherapy (approximately 22 months after treatment initiation), the patient developed worsening dyspnoea requiring emergency hospitalization. On admission, he exhibited severe hypoxaemia (SpO_2_ 91% on 4 l/min oxygen), which worsened markedly with minimal exertion. Physical examination revealed no jugular venous distension, cardiac murmur, or peripheral oedema. Chest CT showed bilateral diffuse ground-glass opacities, mediastinal lymphadenopathy, and pleural effusions, without evidence of new lesions suggestive of pneumonia, recurrent lung cancer, or pulmonary thromboembolism (*Figure 2D*). Additionally, electrocardiography and echocardiography demonstrated signs of right ventricular pressure overload (*Figure 2E, 2F*).

Further investigation revealed a markedly reduced diffusing capacity of the lung for carbon monoxide (20.9%, previously 68.6% at surgery), and an elevated brain natriuretic peptide concentration (498 pg/ml; normal range <18.4 pg/ml; previously 16.2 pg/ml). Right heart catheterization confirmed PH, with a mean pulmonary artery pressure of 43 mmHg, systolic pulmonary artery pressure of 73 mmHg, and pulmonary vascular resistance of 6.7 Wood units. The pulmonary artery wedge pressure was 13 mmHg. Ventilation-perfusion scintigraphy demonstrated minor mismatched perfusion defects, but pulmonary angiography showed no evidence of chronic thromboembolic PH. These findings indicated PH with increased pulmonary resistance and reduced diffusion capacity.

Because pre-capillary PH was initially suspected, selexipag was initiated at 0.4 mg/day along with intensified diuretic therapy. However, the patient’s hypoxaemia rapidly and progressively deteriorated, leading to cardiac arrest and the initiation of veno-arterial extracorporeal membrane oxygenation. He died on hospital day 53.

**Figure 3:**
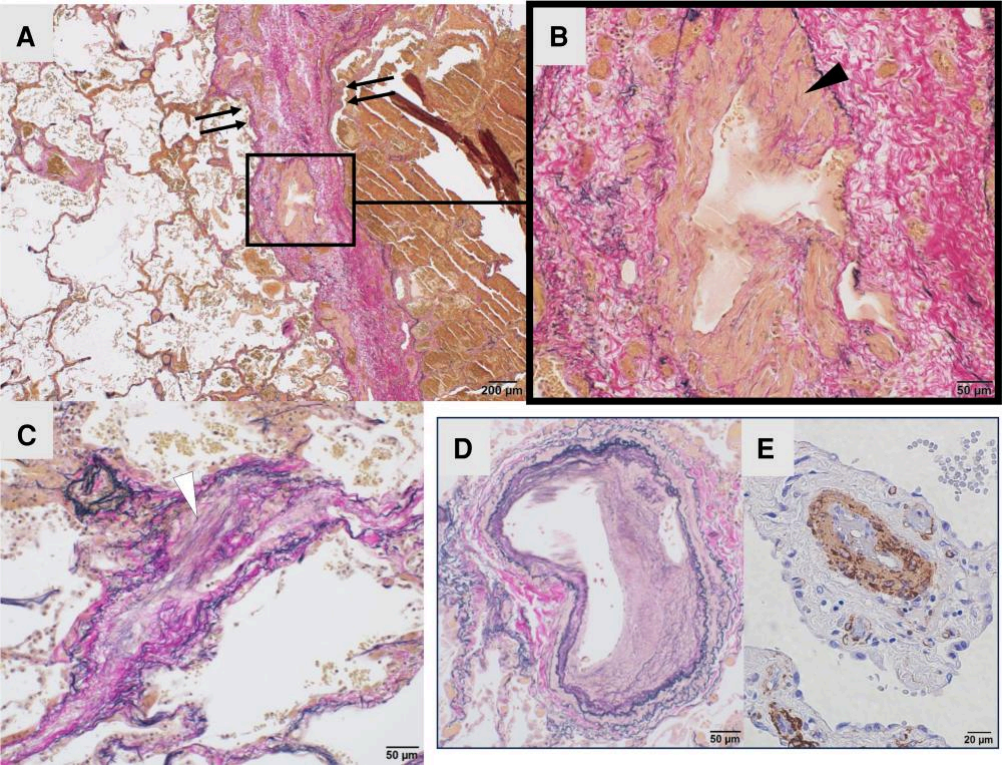
Histopathological findings of the lung at autopsy. (*A*) Histopathological examination reveals interlobular septal thickening (arrows). (*B*) Interlobular pulmonary veins demonstrate smooth muscle proliferation (arterialization) (black arrowhead) and (*C*) marked intimal fibrous thickening (white arrowhead). Pulmonary arterioles exhibit (*D*) intimal thickening and (*E*) medial hypertrophy (muscularization), with no complex plexiform lesions. Panels a–d: Elastica van Gieson staining; panel e: α-smooth muscle actin immunostaining.

Autopsy revealed right ventricular wall thickening (7 mm) and hepatosplenomegaly, consistent with chronic, severe PH. Histopathological examination of the lungs demonstrated interlobular septal thickening and obliteration of the peripheral pulmonary veins, characterized by smooth muscle proliferation and marked intimal fibrous thickening, consistent with PVOD (*Figure 3A–3C*). Although there were mild intimal thickening and medial hypertrophy of the peripheral pulmonary arteries, these changes were less pronounced than the venous remodelling (*Figure 3D, 3E*). There was no evidence of recurrent or metastatic lung cancer, chronic thromboembolic disease, or pulmonary tumour thrombotic microangiopathy. There were no inflammatory changes in the pulmonary vessels. There was mild left ventricular hypertrophy, but no specific pathological findings in the myocardium.

## Discussion

We report a rare case of PVOD that became clinically evident after approximately 2 years of ICI therapy for lung cancer. Although causality cannot be definitively proven, the clinical course and pathological findings suggest that ICI exposure contributed to the development and progression of PVOD. Although PVOD shares clinical and haemodynamic features with PAH and is classified as a distinct subtype of PAH in current guidelines, recognizing venous involvement remains crucial because PVOD differs substantially from PAH in pathophysiology, prognosis, and response to pulmonary vasodilator therapy.^7–9^ As the use of ICIs expands across various malignancies, clinicians should remain vigilant for irAEs, including rare pulmonary vascular complications. This case reinforces the need to consider PVOD as a differential diagnosis in ICI-related PH.

Recent studies suggest a potential immune-mediated mechanism linking ICI therapy to vascular injury.^4,10,11^ It has been proposed that uncontrolled T-cell activation and inflammatory cytokines promote vascular damage and remodelling, as described in ICI-associated vasculitis.^11^ Furthermore, PH is associated with abnormal regulatory T-cell function and dysregulated immune responses.^12,13^ Because regulatory T cells protect vascular endothelial cells, ICIs might contribute to PVOD by enhancing T-cell immune activation, although the specific mechanisms targeting pulmonary veins remain unclear.

In this case, long-term nivolumab administration might have contributed to PVOD progression. Notably, the patient developed respiratory failure and renal irAE shortly after the initial administration of nivolumab and ipilimumab. The new-onset ground-glass opacities observed on CT suggest that PVOD may have started to develop at that time. While other cardiac irAEs, such as myocarditis, typically develop early after ICI initiation, pulmonary vascular toxicity has been reported to correlate with the duration of nivolumab therapy.^3,10,14^

Given the patient’s history of malignancy, chronic thromboembolic PH was considered in the differential diagnosis; however, autopsy demonstrated no pulmonary arterial lesions consistent with this condition, whereas the predominant abnormalities involved the pulmonary veins. Although idiopathic PVOD and an underlying genetic predisposition could not be completely excluded, the pathological differences observed in the pulmonary vasculatures before and after ICI therapy support a temporal association between ICI therapy and PVOD development. Environmental risk factors for PVOD include certain anticancer agents (e.g. alkylating agents) and smoking.^8^ The patient had no exposure to alkylating agents. He had a significant smoking history, and genetic testing, including *EIF2AK4* analysis, was not performed; however, no PVOD-related pathology was identified at the time of lobectomy. These findings support the notion that ICI therapy played a contributory role in the development of PVOD in this patient.

## Conclusion

Clinicians should consider PVOD in patients receiving ICI therapy who develop unexplained PH and severe hypoxaemia. Although differentiation from pre-capillary PAH remains challenging even with invasive haemodynamic testing, awareness of key features such as disproportionately reduced diffusing capacity may facilitate earlier recognition and appropriate management.

## Data Availability

The data underlying this article will be shared on reasonable request to the corresponding author.

## Lead author biography

Saki Hasegawa-Tamba works as a cardiologist at Saitama Medical University, International Medical Center, specializing in adult congenital heart disease (ACHD) and related pathologies, such as advanced heart failure and pulmonary hypertension (PH). Her current research focuses on unique pathophysiology and haemodynamics in ACHD and PH patients.

## Notes

**Consent:** The authors confirm that written consent for submission and publication of this case report, including images and associated text, was obtained from the patient’s spouse in line with the Committee on Publication Ethics (COPE) guidelines.
